# Supplementary material for: Utility of enhanced recovery after surgery protocols in reducing postoperative opioid use across different surgical specialties—An analysis of Iowa’s billion pill pledge program
Source: PLoS One. 2025 Sep 19;20(9):e0332205. doi: 10.1371/journal.pone.0332205 (PMC12448346; doi:10.1371/journal.pone.0332205)
Supplement: Attachment — Submitted filename: Response to Reviewers.docx [file pone.0332205.s001.docx]

Dear Editor-in-Chief of PLOS ONE,

We sincerely thank you for the opportunity to revise and resubmit our manuscript titled:

"Utility of enhanced recovery after surgery protocols in reducing postoperative opioid use across different surgical specialties – an analysis of Iowa’s billion pill pledge program”

We appreciate the thoughtful comments provided by the editorial team and peer reviewers. We have revised the manuscript accordingly and addressed each point as detailed below. All changes have been incorporated into the main text per PLOS ONE guidelines.

Please find our detailed responses below. Thank you.

Reviewer# 1 Comments:

1. Great work at demonstrating that common sense pain practices help in reducing opioid prescriptions and usage.

If you could define the pre-ERAS implementation opioid quantity more granularly that would be good. In your discussion you reported that the surgeons gave you an estimate of how much they would prescribe before. How did they determine that? Did each of the surgeons or their practices have an opioid prescription protocol for individual procedures that they would follow? If they did that would be a much more reliable number than an estimate and you could put that into the manuscript.

Response: We sincerely thank the reviewer for their positive feedback to our manuscript as well as for bringing their concern to our attention. Pre-ERAS prescription sizes used in this study were surgeon-generated estimates of the amount of opioids the patient would have theoretically been prescribed postoperatively had they been treated prior to the launch of the Billion Pill Pledge. Each patient’s pre-ERAS estimate was generated by the respective surgeon in charge of their care. Surgeons based these estimates off their prior prescribing habits for similar procedure types as indicated by hospital records. These pre-ERAS hypotheticals were input into a deidentified database of surgical patients cared for at BPP partner sites. It was this database that was retrospectively screened for the purpose of this study. The portion titled “Measuring opioid quantities” under our methods section has been revised to better illustrate this workflow.

2. How did you retrospectively get patient reported measures regarding pain medication usage on a post op visit? When you said you validated that with the providers is it all from chart review? If the Billion Pill Pledge enrollment required documentation enhancement to be able to reliably capture that data it would be wise to mention that in the manuscript.

Response: We thank the reviewer for bringing this to our attention. Upon initiation of the Billion Pill Pledge, standing orders at all partner sites were updated to instruct nursing staff to prospectively collect opioid prescription and consumption data via patient follow up over the phone. This data was documented into a deidentified database, which was retrospectively screened for the purpose of this study. The portion titled “Measuring opioid quantities” under our methods section has been revised to better illustrate this workflow.

3. In figure 1- are all ERAS protocols the same for any and all surgeries barring what your mentioned? Is celecoxib/meloxicam a one time use before surgery? In other words a patient getting a knee replacement will get the same 10 dose opioid script as someone who had a diagnostic arthroscopy? Also either use Tylenol or Acetaminophen (just a minor detail)

Response: We thank the reviewer for highlighting their concerns. Upon initiation of the Billion Pill Pledge, standing orders at each partner site were updated to implement this pathway for all surgeries performed. Exceptions were made only as necessary, at the surgeon’s discretion. While celecoxib/meloxicam use is noted as a “Before Surgery” intervention, it may also be used as the selective Cox-2 NSAID of choice for pain management “After Surgery”, as per physician’s discretion. This has been listed in the “Implementation of billion pill pledge/ERAS pathways” portion of the methods section. We have also made revisions to clarify that each patient was limited to a maximum of 10 opioid doses. While different patients may have been prescribed different quantities of opioids, the maximum quantity prescribable as per updated standing orders was 10 doses. This opioid script was meant to be reserved for severe breakthrough pain, while the first line of pain management postoperatively focused on non-opioid medications. We have also revised the manuscript to replace “Acetaminophen” with “Tylenol”.

4. Could you get a breakdown of inpatient versus outpatient surgeries on your dataset? If inpatient surgeries how did you capture post op opioid use while the patient was hospitalized. Did you have access to their inpatient medication administration record? What was the average length of stay of inpatient surgeries?

Response: We thank the reviewer for sharing their concerns. In compliance with the Billion Pill Pledge, all surgeries were performed in the outpatient setting. The deidentified database screened in this study therefore does not contain any inpatient medication records. The purpose of this study was to track only postoperative opioid medications made at discharge (or after). We have revised the methods section to more explicitly state this.

5. You have a breakdown of the kind of surgeries that were performed, can we get MMEs associated with each of the individual surgeries broken down in a table instead of Orthopedics vs General Surgery? Similarly if you have data for left over opioids for each of these procedures, you may be able to predict which type of surgical procedure patients need a full 10 dose script versus who doesn't?

Response: We thank the reviewer for bringing this concern to our attention. The deidentified database screened in this study did indeed include information on procedure type. However, we were unable to acquire cohort sizes large enough to perform a statistically powered comparison between all procedure types. Recognizing the value of a statistically powered analysis, we decided to base the study instead on a Cross-specialty comparison. We have acknowledged this constraint in the limitations paragraph of our discussion section. We have also revised the methods section to more explicitly present the findings of our a priori analysis.

6. Finally, does the dataset capture prior history of opioid use or chronic opioid use in the patients?

Response: We thank the reviewer for highlighting their concern. Unfortunately, the database screened in this study contains no patient information other than procedure type, opioid prescriptions, and opioid consumption data. This prevents the study design from accounting for opioid consumption habits in opioid naïve patients versus chronic opioid users. We have included this constraint as one of the limitations of this study in the discussion section.

Journal Requirements

1.“The data underlying the results presented in the study are available from Goldfinch Health Incorporated, who can be contacted at https://www.goldfinchhealth.com/”

All PLOS journals now require all data underlying the findings described in their manuscript to be freely available to other researchers

Response: We thank the reviewer for sharing their concern. We have attached a minimal data set among our supplementary documents uploaded. This document includes the means and standard deviations used to create the tables and graphs within this manuscript. These statistics were calculated using data provided to our non-profit research foundation by a third party: Goldfinch Health Incorporated. Given that the authors of this manuscript do not have permission to share this third party data, we urge interested researchers to contact the Vice President of Program Success at Goldfinch Health Inc, Meredith Allan at [Meredith.Allan@goldfinchhealth.com](mailto:Meredith.Allan@goldfinchhealth.com)

Interested researchers may replicate our study findings entirely by directly obtaining data from Goldfinch Health Inc and following the methodology outlined in our manuscript.
